# Safe and effective short-time percutaneous cholecystostomy: A retrospective observational study

**DOI:** 10.1097/MD.0000000000031412

**Published:** 2022-11-04

**Authors:** Miroslav Šimunić, Liana Cambj Sapunar, Žarko Ardalić, Marin Šimunić, Dorotea Božić

**Affiliations:** a Department of Gastroenterology and Hepatology, University Hospital Split, Split, Croatia; b Department of Diagnostic and Interventional Radiology, University Hospital Split, Split, Croatia; c Department of Haematology, University Hospital Split, Split, Croatia.

**Keywords:** acute cholecystitis, PCT drainage, percutaneous cholecystostomy

## Abstract

The introduction of percutaneous cholecystostomy (PCT) has shifted the paradigm in treatment of acute calculous and acalculous cholecystitis. PCT has high success and low complication rates, but there are still unresolved issues regarding the duration of the procedure. The aim of our study is to determine the characteristics and outcome of patients treated with short-term PCT drainage. Patients who were admitted to the Department of gastroenterology and the Department of Abdominal Surgery at the University Hospital Center Split under the diagnosis of acute cholecystitis and who were treated with the PCT, in a period between January 2015 and January 2020, were retrospectively included in the study. During that timeframe we identified 92 patients and have analyzed their characteristics and clinical outcomes. The statistical analysis included the Kaplan–Meier method for calculating survival curves for grades 2 and 3, the log-rank test for testing the difference between survival rates of grade 2 and 3 patients, and logistic regression to determine variables that affected the outcome of our patients. According to the Tokyo guidelines, most of the patients (74, 80.43%) met the criteria for grade 2 cholecystitis, and the minority had grade 1 (9, 9.78%) and grade 3 (9, 9.78%) cholecystitis. The average drainage duration was 10.1 ± 4.8 (3–28) days. We identified mild complications in 6 cases. Nine patients (10%) had lethal outcome. The mortality in the largest group of patients with grade 2 cholecystitis was 5.48% and as high as 71.43% in patients with grade 3 cholecystitis. The complication rate was 6.5%. One quarter of gallbladder aspirates showed a ciprofloxacin resistance. Short-time PCT lasting approximately 10 days can be used safely and effectively for the treatment of patients with acute cholecystitis.

## 1. Introduction

The introduction of percutaneous cholecystostomy (PCT) has shifted the paradigm of treatment for acute calculous and acalculous cholecystitis. PCT decompresses the gallbladder, thereby facilitating the migration of the gallstones that usually obstruct the cystic duct, and consequently reduces inflammation. First PCT was described in 1921. as a radiological exam and was introduced for therapeutic purposes in patients with gallbladder empyema by Radder.^[1]^ Thanks to enhanced technical possibilities and emerging experience in the application of this method it is currently widely used in those patients for whom the surgical approach is too risky due to comorbidities, or with contraindication for general anesthesia.

According to Tokyo guidelines 2018 (TG18), we can differentiate between three stages of severity in acute cholecystitis (AC).^[2]^ Patients with grade 1 AC should be treated either conservatively or with early laparoscopic cholecystectomy (Lap-C). The latter modality is also the treatment of choice for patients with grade 2 AC that meet following criteria: Charlson Comorbidity Index (CCI) ≤ 5 and American Society of Anesthesiologists physical status classification system ≤ 2. In patients with high risk for surgical treatment due to severe comorbidities, or grade 3 AC, PCT should be used as a bridge to interval cholecystectomy, as well as definitive treatment.^[2,3]^ Reported efficacy rates prove that PCT can be used as final treatment modality, with low rates of recurrent disease and without a need for interval cholecystectomy in a significant number of patients.^[4,5]^

PCT has demonstrated high success rates of approximately 90%^[4,6]^ and low complication rates,^[6]^ but there are still unanswered questions regarding the procedure timing and duration. Studies suggest that an early PCT (≤24 hours) reduces the length of hospital stay and procedure-related bleeding rate, with an intrahospital mortality rate of approximately 7%.^[7]^ Regarding PCT duration, it has become recognized that the prolonged PCT is related to the extended hospital stay, higher risk of tube disfunction and PCT related complications, as well as patient discomfort. The aim of our study is to determine the patient characteristics and intrahospital outcome of patients treated with the short-term PCT, including clinical response, complication development and mortality.

## 2. Materials and Methods

Patients who were admitted to the Department of Gastroenterology and the Department of abdominal surgery at the University Hospital Center Split under the diagnosis of AC, and who were treated with the PCT drainage, in a period between January 2015 and January 2020, were retrospectively included in the study. During the observed period we identified 92 patients. Patients with liver abscesses treated with percutaneous drainage were not included in the study.

**Figure 1.**
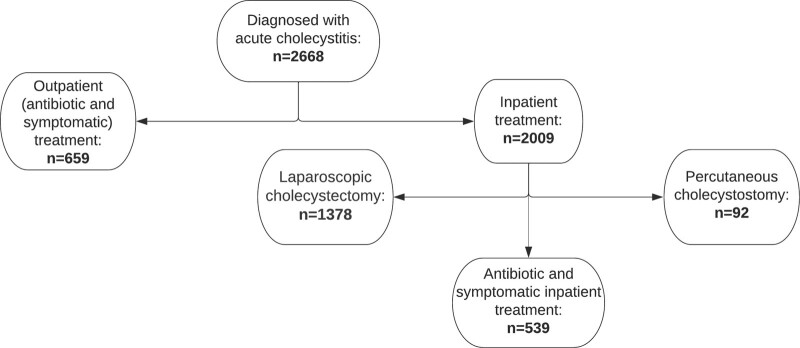
Flowchart presenting patients examined at the emergency unit of University Hospital Center Split from January 2015 until January 2020.

Flowchart is presented as Figure 1.

All procedures were performed in accordance with the ethical standards of the institutional and national research committee and with the 1964 Helsinki declaration and its later amendments or comparable ethical standards. Ethical approval was waived by the local Ethics Committee of University Hospital of Split due to the retrospective nature of the study and since all the procedures being performed were part of the routine care. The datasets generated and analyzed during the current study are available from the corresponding author on reasonable request.

The primary study endpoint was intrahospital outcome of patients treated with PCT, defined as: survival, death, and referred to urgent cholecystectomy. The secondary endpoints were the influence of age, comorbidities (defined by the CCI), laboratory parameters, delay in the drainage initiation and drainage duration on the patient outcome; as well as the complication assessment. The most commonly isolated bacterial strains and their antibiotic susceptibility to the first line empiric antibiotic treatment (ciprofloxacin) have also been investigated.

### 2.1. Sources of data

Data was collected from hospital records of patients referred to PCT at the Department of Clinical Radiology at the University Hospital Center Split.

The patient records were searched for: age, sex, grade of AC according to TG18, laboratory parameters on day 1, day 3 and before discharge (leukocytes, creatinine, C-reactive protein, total bilirubin, platelets), CCI, the choice and duration of antibiotic therapy, timing and duration of drainage, length of hospital stay, as well as complication development.

### 2.2. Method

PCT was performed using Seldinger technique. Under aseptic condition and after local anesthesia the puncture of gallbladder via transhepatic access route under CT guidance was performed. Aspirated fluid was collected for microbiology analysis. 8-10 French (catheter size) drainage catheter (Abscess Drainage Set, Optimed, Ettlingen, Germany) was placed and secured on the skin for continuous drainage of infected bile. Drain was flushed daily with 2 × 10 mL of saline to prevent drain clogging.

Drain removal was performed at the discretion of the physician, when the patient no longer showed signs of infection and when the bile collected in the drainage bag was normally colored.

### 2.3. Statistical analysis

The statistical analysis was performed with the Windows software (version 22; IBM, Armonk, NY). Significance was defined as *P* < .05. Data are expressed as mean and standard deviation. Descriptive statistics were computed for characteristics of patients and laboratory data. Survival curves for grades (grade 1 was omitted since there were no deaths in that group) were generated by the Kaplan–Meier method. The log-rank test was used to test the difference between survival rates of grade 2 and 3 patients. Survival rates are shown in % with 95% confidence intervals. In order to determine variables that affected the outcome of our patients, we used binary logistic regression (patients referred to urgent cholecystectomy were excluded from that analysis). The analysis was carried out by computing odds ratios and their 95% confidence intervals to compare outcomes defined as either death or hospital discharge for each potential risk factor of interest.

## 3. Results

### 3.1. Patient characteristics

We have included 92 patients in the analysis. There were 47 (51.08%) males, and the mean age was 76.5 ± 10.1. Almost all patients had pericholecystitis (95.65%), as well as calculi as the cause of inflammation (89.01%). Only 19.57% of patients had gallbladder necrosis, wall emphysema or the consequent liver abscess formation. One third of patients (32.61%) suffered from gallbladder perforation.

According to TG18, most of the patients (74, 80.43%) met the criteria for grade 2 AC, and the minority had grade 1 (9, 9.78%) and grade 3 (9, 9.78%) AC.

### 3.2. Isolated strains and antibiotic treatment

The list of isolated strains from the gallbladder aspirate is presented in Table 1.

**Table 1:** The number of isolated strains from the gallbladder aspirate.

Bacterial strain	Count
Escherichia coli	19
Streptococci	15
Klebsiella	16
Sterile culture	20
Enterococcus	9
Staphyloccoci	10
Enterobacter	9
Proteus	3
Acinetobacter	1
Candida albicans	2
Pseudomonas	2
Serratia	2
Clostridium perfrigens	1
Bifidobacterium	1

Among 81 patients whose cholecystic fluid underwent microbiological examination, 20 had sterile culture, 40 patients had one isolate, 15 of them had 2 isolated species and 6 patients had 3 various isolates. Among them 8 patients had lethal outcome and they were classified as follows: 2 (2.47%) had sterile culture, 2 (2.47%) had 1 isolate, 3 (3.7%) had 2 isolated species and only 1 deceased patient (1.23%) had 3 different isolates. The number and type of isolated bacterial strains had no effect on the outcome.

Regarding the antibiotic treatment, majority of patients were treated with ciprofloxacin (78.26%) and metronidazole (92.39%), and 65 of them (70.65%) with the combination of the aforementioned antibiotics, while triple antibiotic therapy regimen was used in 8 patients only. Fifteen patients were treated with meropenem or ceftriaxone, while other antibiotics were used in a quite small percentage.

Among 56 gallbladder aspirates tested for ciprofloxacin susceptibility, 14 of them (25%) showed ciprofloxacin resistance. In 34.83% of patients, therapeutic regimen has been changed after the antibiogram arrival. The average duration of antibiotic treatment was 15.5 ± 7.3 (5–45) days. Using the binary logistic regression we did not find the duration of antibiotic treatment influencing the patient outcome. The difference between survival rates in patients depending on their ciprofloxacin resistance (chi-square = .892, *P* = .345) or change in the antibiotic regimen (chi-square = 6.567, *P* = .010) was not significant.

### 3.3. Laboratory parameters and comorbidities

**Figure 2.**
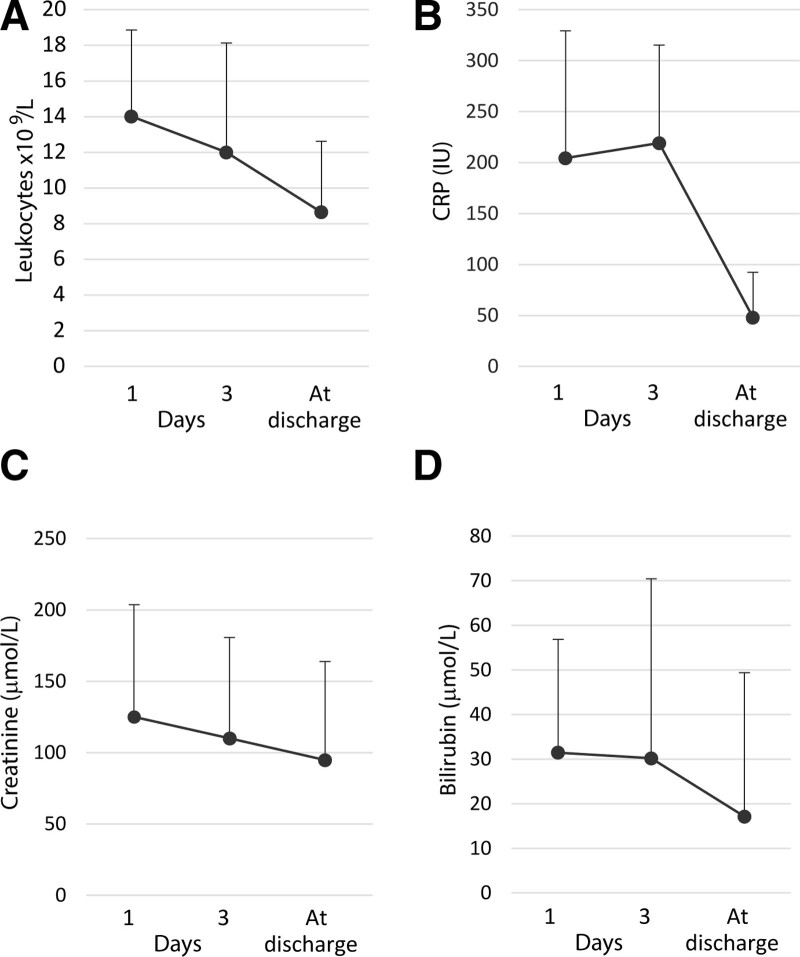
Dynamic of laboratory parameters in patients treated with short-term PCT from day 1 until discharge. PCT = percutaneous cholecystostomy.

The change in laboratory parameters (leukocytes, creatinine, C-reactive protein, total bilirubin, platelets) depending on their values measured on day 1, day 3 and before discharge is presented graphically in Figure 2.

The average CCI was 4.52 ± 1.82, and as expected, higher than in general in patients with grade 3 AC (5.88 ± 1.55).

### 3.4. PCT timing and duration

**Figure 3.**
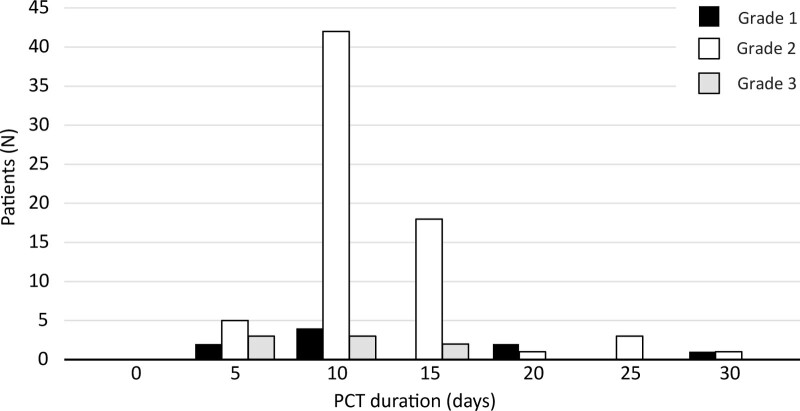
Duration of PCT depending on the grade of cholecystitis. PCT = percutaneous cholecystostomy.

The average hospitalization length was 15.1 ± 7.6 (5–45) days, similar as the duration of the antibiotic treatment. The average drainage duration was 10.1 ± 4.8 (3–28) days. In patients with grade 2 AC, PCT lasted for 10.1 ± 4.3 days. The shortest duration was documented in patients with grade 3 AC (7.6 ± 3.7 days), probably due to high percentage of lethal outcomes (Fig. 3). The average timing for drainage initiation was 6.24 ± 4.6 (1–20) days.

Only six patients developed postprocedural complications including: gallous secretion beside the drain (1 patient), reapplying the drain due to malposition or blockage (2 patients), cellulitis (1 patient) and the drain fall-out (2 patients). There were no reported severe adverse events.

### 3.5. Outcomes

Among 90 patients with registered outcome, 75 (83.33%) of them were discharged after the clinical improvement, 6 (6.67%) patients underwent urgent cholecystectomy with favorable outcomes and 9 (10%) patients had lethal outcome.

**Figure 4.**
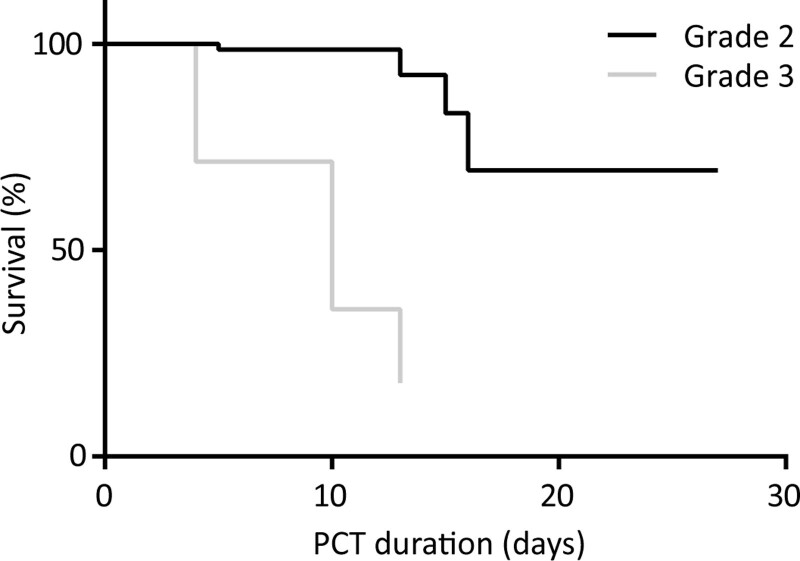
Kaplan–Meier curve depicting the survival of patients listed as grades 2 and 3 following PCT. PCT = percutaneous cholecystostomy.

**Figure 5.**
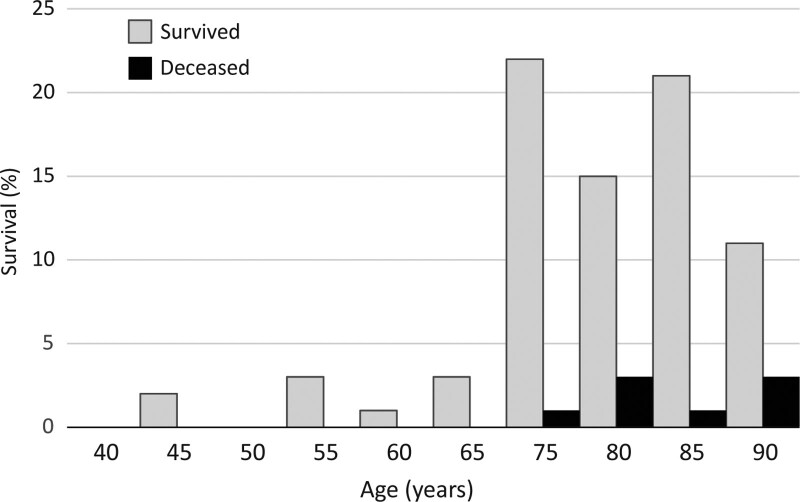
Age dependent distribution of patient outcome.

Among the 9 patients with grade 1 AC, there were no lethal outcomes, and only 1 patient underwent urgent surgery. Four patients died in the group with grade 2 AC, and 5 patients out of 7 with registered outcome (71.43%) in the group with grade 3 AC. We must emphasize that the mortality in the largest representative group of patients with grade 2 AC was 5.48% and 71.43% in the most severely ill group of patients with grade 3 AC (Fig. 4). The difference between survival rates in patients belonging to AC 2 and AC 3 was significant (chi-square = 36.7, *P* < .001). It is also important to notice that all of the lethal outcomes occurred in patients over 75 years of age. Age dependent distribution of outcome is presented in Figure 5.

The binary logistic regression preformed in order to determine variables that predict negative outcome of the patients following the procedure did not reveal any factor significantly contributing to the death of patients.

## 4. Discussion

PCT is a safe and effective alternative to emergency cholecystectomy for patients with AC and comorbidities that pose too great surgical or anesthetic risk. Without doubt, it leads to clinical and radiologic improvement, as well as better surgical outcomes in interval cholecystectomy. The adequate selection of patients to be treated with this method is highly important, and the decision on transhepatic or transperitoneal access depends on the patients’ comorbidities and radiologists’ equipment and experience, while both approaches have apparent advantages and disadvantages. The transhepatic method is performed by approaching the liver-adhering areas of the gallbladder wall, and it allows for better fixation of the drainage catheter in the penetrated liver. The transperitoneal access on the other hand is the method of choice in patients with liver diseases or coagulopathies, as it approaches the gallbladder wall covered with the visceral peritoneum.^[8]^ The patients in our cohort have all been treated with the transhepatic approach, according to the discretion and experience of our interventional radiologists.

### 4.1. Antibiotic treatment

Antibiotics are a very important part of the treatment of patients with AC who qualify for, or have undergone, a PCT. In patients with grade 1 AC, they have a predominantly preventive role, while in grade 2 and 3, they are indispensable as a support to the PCT. TG18 state which empirical antibiotic therapy should be applied, depending on the degree of AC, comorbidities, allergies, previously used antibiotics, renal and hepatic function, and local antibiotic resistance. Referring to 2013 Tokyo guidelines and due to increasing bacterial resistance to beta-lactams and fluoroquinolones, they are no longer recommended for milder forms of AC, if the locally reported resistance rate is greater than 20%.^[9,10]^ As expected, *Escherichia coli* (36 patients) and Klebsiella (27 patients) were detected as the most common causes of infection in bile isolates in our cohort of patients (Table 1). In our study conducted between January 2015 and January 2020, fluoroquinolones and metronidazole were the most commonly used empirical therapies for patients with AC 1 and AC 2, in accordance with previous attitudes and local resistance patterns in our region, as well as the recommendations to treat patients with broad-spectrum antibiotics before the percutaneous intervention. Proven resistance to fluoroquinolones of 25% in bile isolates obtained by PCT in our patients, warns that fluoroquinolones as empirical therapy are to be replaced by second- or third-generation cephalosporins, with or without metronidazole, as well as carbapenem-based therapies.

### 4.2. PCT initiation

In previous studies, the indications for PCT placement in patients with high surgical or anesthetic risks for intervention were: failure to respond to medical treatment, severe systemic inflammatory response syndrome, suspected or menacing empyema/necrosis/perforation of gallbladder, patient refusing surgery, surgeons’ discretion, and advanced age. The most common cause was failure to respond to medical treatment with intravenous antibiotics, but there was no consensus regarding the exact duration of antibiotic treatment and optimal time of PCT insertion,^[11]^ so it varies from 6 hours to 77 days in different studies due to conflicting local guidelines.

Although there is no consensus, it is evident that the drainage initiation should be performed as soon as possible, preventing the progression of inflammation, the formation of adhesions and the adjacent tissue damage. In a retrospective analysis of 271 patients, Noh et al^[8]^ demonstrated significant withdrawal of symptoms and an improvement in laboratory findings in 86.7% of patients 4 days after the intervention, while by placing drainage early, within a day, Chou et al^[7]^ were able to achieve shorter hospital stays and fewer complications.

When comparing early- with the delayed PCT group at the time of subsequent cholecystectomy, Bickel et al^[12]^ reported lower rates of conversion to open surgery (8.3% vs 33.3%, *P = *.09) and a considerable difference in the level of intraoperative adhesions (25% vs 57%, *P = *.064) in the early group. In our study, the time to PCT setting was on average after 6.24 ± 4.6 (1–20) days, which can and should be shortened in accordance with the results and recommendations of recent studies showing obvious efficacy and safety of the early intervention. Due to the lack of standardized criteria, Chok et al^[13]^ indicated PCT if there was no improvement in clinical status or laboratory parameters after only two or three doses of empirical treatment with cefuroxime.

### 4.3. PCT duration

The aim of our study was to give the yield into the decision making regarding the duration of PCT which varies significantly between the Centers. Some recent studies recommend later catheter removal which allows the maturation and prevents bile leakage, recurrent AC attacks, and readmissions. Hasbacceci et al^[14]^ recommend keeping the PCT tube in place until the time of surgery in surgically fit patients, as well as leaving it in place as long as possible in patients unfit for operation or general anesthesia. Also, they consider safe the elective removal of a cholecystostomy tube after the tract has matured and cholecystitis has resolved, which usually takes 3 to 6 weeks after placement.^[14]^ In a large study on 324 patients mean cholecystostomy tube indwelling time was 89 days (range 0–586 days),^[6]^ similar to results shown in a study on 89 patients (average PCT duration 64.1 ± 37.4 days) conducted by Tullius et al.^[15]^

However, the vast majority of authors have proposed recently that the PCT tube should be removed after the resolution of AC.^[16–18]^ A systematic review of 50 studies revealed that the timing of tube removal varied from 1 to 427 days, with no significant difference in overall outcome depending on the duration of PCT.^[17]^ Analyzing the impact on mortality, morbidity and recurrent disease, the authors showed no correlation related to the PCT indwell and therefore concluded that there was no evidence on whether the PCT duration might affect the outcome. Di Martino et al^[18]^ confirmed in the study on 151 patients that early removal of the PCT catheter did not cause an increased risk of biliary leakage or peritonitis, neither in the transhepatic nor in the peritoneal approach. Their policy was the early drain removal once the patient presented a low biliary output in the absence of deranged liver function tests, especially in subjects who were potential candidates for a delayed cholecystectomy. Comparing patients who had their drain removed during their hospital stay after an average of 8 days (6–11), with discharged patients who had their drain removed after 52 days (26–67), no difference was seen regarding recurrent disease rate and readmissions. The study showed that in cases of drain removal within the 7 days, the duration of antibiotic treatment was significantly shortened, but those patients were at increased risk of recurrent disease and readmission.^[18]^

In our institution, we also preferred earlier PCT tube removal after AC resolution evaluated by clinical examination and laboratory parameters of inflammation. The average drainage duration was 10.1 ± 4.8 (3–28) days, and only in 9 patients (9.8%) it lasted more than 15 days. In patients with grade 2 AC, drainage lasted similarly to the overall average, and shorter drainage duration in the group of patients with grade 3 AC was caused by a higher mortality rate due to AC severity and comorbidities. The overall hospitalization length was 15.1 ± 7.6 (5–45) days, depending mainly on the duration of antibiotic treatment before and after the intervention.

### 4.4. Complications

As predicted by a wide variety of studies, the incidence of PCT-related complications varies between 2.5% and 69%.^[19]^ Fortunately, the most commonly reported complication is the catheter displacement (more than 50% of complications).^[19,20]^ Bile leakage is another common adverse event,^[20,21]^ whereas events such as catheter obstruction, infection, bleeding, organ perforation and lethal outcome are relatively rare.^[20,22]^ Complication management approach varies and is usually individualized. Venkatanarasimha et al^[23]^ published a set of principles for diagnosis and treatment of biliary intervention complications, including PCT procedures. Hung et al^[19]^ cited their own experience as a recommendation, as there are currently no widely accepted guidelines, and they recommend individual assessment for patients with complete PCT tube displacement. Once the patient is confirmed to be asymptomatic, he may be discharged without re-PCT. For patients in whom partial displacement of the tube is suspected, cholangiography may be used to confirm the position. The decision to maintain or remove drainage tubes is made by physicians or radiologists based on the general condition of the patient. In case of bile leakage, drainage under the control of one of the imaging methods with antibiotic support is recommended. Minor hemorrhages are most commonly treated conservatively, and major ones by spiral embolization or surgically. Patients with suspected bile duct obstruction should undergo a cholangiography and, according to its finding, a PCT should be reinstated or a cholecystectomy performed, depending on the patient’s clinical condition. In our study, only six patients developed postprocedural complications including drain displacement, biliary secretion beside the drain and cellulitis. All of the complications were successfully treated conservatively during the hospital stay and did not require urgent surgery or suffer a significant deterioration in general condition or a fatal outcome.

### 4.5. Outcome

When considering short term outcomes, published studies have reported a rate of 5.4% to 15%, similar to the outcome of our patients that was 10%.^[7,8,13,16,22]^ We have detected statistically significant difference in the mortality of patients regarding the stage of inflammation: 5.48% in patients with grade 2 AC and 71.43% in patients with grade 3 AC. The reason for this discrepancy is the higher grade of inflammation, older age and higher comorbidity rate in the latter group. Other authors have found septic condition and comorbidities to be the most common cause of death.^[7,8,13,16,22]^ We have not found a significant difference in the outcome depending on the drainage initiation or duration.

When considering long term outcomes, Loozen et al^[24]^ demonstrated a significant advantage of Lap-C over PCT in 142 high risk patients with AC. According to their study, there was no difference in the survival, but they reported a statistically significant difference in the major complications rate (12% in the Lap-C group and 65% in the PCT group, RR 0.19, 95% confidence interval 0.10–0.37; *P* < .001). This is in accordance with TG18 that state how an early Lap-C should also have priority over delayed cholecystectomy, even within 1 week of the disease, since this has proved to reduce hospital stay and costs, with lower chance for additional treatment requirements.^[2]^

It is important to distinguish that mortality in the group with grade II cholecystitis was only 5.48%, and very high (71.43%) in patients with grade III cholecystitis. Patients in both groups had high Charlson indices (≥5 in TG II and 6–8 in TG III), which in itself is marked by a high mortality risk. All patients with fatal outcomes were over 75, and three were over 85 years of age. Old age, high APACHE II and CCI score, and TG grade III at the beginning of treatment are unquestionably poor prognostic parameters of mortality in PT. Lower mortality and increased efficacy of PT can be achieved by avoiding procedural complications such as bleeding, perforation, and stent migration or contamination. In patients who do not respond to adequate conservative treatment, PT should be performed as soon as possible, preferably within 48 hours, preferring the transhepatic route. The drainage duration is a matter of judgment in each individual case, with increasing evidence as of recently, proving that shorter drainage duration produces better results. In addition to general measures and empiric antibiotic therapy, of utmost importance are microbiological testing and the application of the most effective therapy, matched according to the sensitivity of the pathogens detected in the drained sample. Cholecystectomy should be considered in all patients whose general condition and local inflammation are significantly reduced.

Therefore, early Lap-C obviously shows abundant cost-related and long-term benefits, and should unambiguously make the first choice of treatment, even in the group of high-risk patients. On the other hand, a clinician should always approach the patient on the individual basis, accept their treatment choices, remain vigilant with the older and severely ill patients, and judicious when assessing the clinical scenario of each patient. The availability of experienced surgeons is also of utmost importance. However, if the mentioned circumstances dictate against the surgical treatment, short-term PCT, along with the antibiotic therapy adjusted to local antimicrobial susceptibility, should be the treatment of choice.

## 5. Conclusion

PCT has become recognized as a valuable method in the treatment of patients with AC in whom the antibiotic treatment is insufficient, and who are not adequate candidates for surgical treatment due to long duration of inflammation or severe comorbidities, or where the treatment of experienced surgeons is not available. Short-term PCT has an advantage over long-term drainage due to shorter hospital stay and reduced costs, while simultaneously not conferring the increased risk for complication development. Short-time PCT lasting approximately 10 days can be used safely and effectively for treatment of patients with AC.

## 6. Limitations

We have not evaluated the long-term complications and outcomes of patients. The retrospective nature of the study bypassed a strictly defined indication for PCT, therefore selected patients had various degrees of inflammation (AC grade 1–3) and had a PCT indication on individual basis.

Further prospective studies evaluating the short-term PCT and its comparison with other treatment modalities in a strictly defined cohort of patients are warranted.

## Acknowledgments

We would like to thank Prof Damir Sapunar for the statistical analysis and the visualization of the Manuscript, his assistance is greatly appreciated.

## Author contributions

**Conceptualization:** Miroslav Simunic, Liana Cambj Sapunar, Zarko Ardalic, Dorotea Bozic.

**Data curation:** Zarko Ardalic.

**Investigation:** Liana Cambj Sapunar.

**Methodology:** Liana Cambj Sapunar.

**Supervision:** Miroslav Simunic, Dorotea Bozic.

**Validation:** Miroslav Simunic, Liana Cambj Sapunar, Zarko Ardalic, Marin Simunic, Dorotea Bozic.

**Writing – original draft:** Miroslav Simunic, Dorotea Bozic.

**Writing – review & editing:** Miroslav Simunic, Marin Simunic, Dorotea Bozic.
